# Editorial: Tennis: Testing and performance

**DOI:** 10.3389/fspor.2023.1190917

**Published:** 2023-04-03

**Authors:** Miguel Crespo, Rafael Martinez-Gallego

**Affiliations:** ^1^ Development Department, International Tennis Federation, London, United Kingdom; ^2^ Faculty of Sciences of Physical Activity and Sport, University of València, València, Spain

**Keywords:** racket sports, evaluation, assessment, tests, tennis

**Editorial on the Research Topic** Tennis: Testing and performance

The topic of testing has attracted considerable attention in the tennis performance literature (1, 2), and the energetic and skill demands of tennis have also been studied extensively providing a considerable understanding of the game challenges (3). In this context, the main objective of this research topic is to assist in the provision of insight on this area by including a series of novel studies that contribute to the acquisition of further theoretical and practical knowledge which can offer new perspectives for research as well as a direct application on-court.

Crucial to the testing process is the use of normative values as done by Johansson et al. who explored the shoulder rotation strength and range of motion of boys’ and girls' junior players and presented a normative database for these aspects. This seminal study showed age and sex differences in both the isometric internal rotation, weakness in external rotation, as well as eccentric external rotation in these competitive athletes. Following the findings from Reid and Schneiker (4) this research emphasised the relevance of strength development through puberty specifically in the girls' tennis players.

The use of technology is gradually becoming a must in the assessment of tennis players (5). However, its application in the provision of information related to the psychological components of the game is still in progress due to the complex nature of mental skills identification in competitive settings. This trend is clearly shown in the study conducted by Havlucu et al. who explored the detection of the achievement of the “zone” in elite players using off-the-shelf wearable technology. They used a neural network to predict the achievement of this state of optimal performance at a relatively low cost. Their findings were successfully applied in a real-life scenario combining wearable technology, expert labels, and machine learning, which could provide all interested with a suitable alternative to the detection of psychological states in tennis players.

The tactical and technical skills constitute the backbone components of the game (6), for the first time ever, Kolman et al. developed a reliable, valid, and feasible tool to assess tactical skills during training and game situations in tennis players. This study supported the psychometric properties of the Tactical Skills Questionnaire in Tennis (TSQT), an instrument by which players can self-assess their tactical skills using reflection on their tactical competencies. This tool can provide useful information to generate specific areas to be worked on during practice sessions. Regarding the testing of the technical aspects, one crucial element in the understanding of the performance of the technical skills in complex match situations. Kolman et al. also explored the development of these skills throughout the various age categories in junior talented players. They used a dedicated tool, the on-court Dutch Technical-Tactical Tennis Test, to find out that skills such as ball speed for males and accuracy in complex situations for both genders develop through adolescence in these players.

As Baiget et al. (7) indicated, the testing of the metabolic and conditional demands of the game provides an unvaluable insight on the profile of this activity. Several articles in this research topic have covered this area. In a systematic review with meta-analysis paper, Lambrich and Muehlbauer quantified and characterised effects of training on physical fitness measures and stroke velocity in youth and adult tennis players. They found training adaptations in adult players for lower-extremity muscle power, upper-extremity muscle strength, and stroke velocity, and endurance adaptations in youth players.

In an applied study, Bjorklund et al. explored the energy expenditure and exercise intensity in four different on-court drills in the line of Reid et al. (8). They concluded that the drills analysed in the study provided energy expenditure per minute values closer to those of similar sports, and that the energy expenditure per meter was considerably greater.

Fatigue is also a capital issue in the tennis testing scenario as stated by Hornery et al. (9). In this context, the relationship between player fatigue and groundstroke type in tennis was studied by Murata and Naito who analysed the physiological demands in simulated matches and hitting tests while considering the translational and rotational kinetic energy ratio of the ball. Findings suggest that players with high lactate levels hit the ball with a greater ratio of rotational kinetic energy to total kinetic energy, which may imply that the type of groundstrokes played is a factor to be considered when testing fatigue in tennis.

The assessment of movement skills in performance players is also extremely relevant due to the great importance of the displacement and court positioning capabilities in today's game (10). In this vein, Reiner Volk et al. explored the correlation of a linear sprint and change of direction test to the current tennis ranking of the player. The authors found that the change of direction test had a moderate and higher impact on tennis performance as compared to the linear sprint. Considering these results, the use of specific change of direction drills in on-court training session was recommended.

From a training programme perspective, the study of the effects of training prescription is extremely relevant (11). Le Sollliec et al. investigated the impact of an 8-week multimodal programme on the thoracic posture, glenohumeral range of motion and serve performance in competitive young players. The programme produced moderately relevant effects to rectify the sagittal thoracic curvature in the sample. It also assisted in regaining the range of motion in glenohumeral rotation without producing an impairment in the performance of the serve.

In conclusion, findings from the articles included in this research topic provide researchers with directions for future studies and facilitate coaches and other professionals with evidence-based methods, procedures and instruments for testing and assessing different components and skills needed for performance tennis. The information presented in this topic can be used in tennis specific testing programmes aimed at increasing the understanding of the mechanisms underlying proper player assessment and evaluation.
